# The voice of a group of teachers in full-service schools in South Africa

**DOI:** 10.4102/ajod.v13i0.1134

**Published:** 2024-02-28

**Authors:** Anna J. Hugo, Nafiza Mobara

**Affiliations:** 1 Department of Language Education, Arts and Culture, College of Education, University of South Africa, Pretoria, South Africa

**Keywords:** full-service schools, teachers’, opinions, support from management, teaching methods, language, religion

## Abstract

**Background:**

Full-service schools (FSSs) were introduced into the South African school system as part of the movement towards inclusive education.

**Objectives:**

The authors of this article embarked on a study to obtain the opinions and feelings of a group of teachers about FSSs and inclusive education. Thus, the ‘voice’ of these teachers could be heard.

**Method:**

The research followed a qualitative approach using group discussions and classroom observations. Triangulation was used to verify information.

**Results:**

The data revealed that the teacher participants had definite opinions and feelings about inclusive education in FSSs and their classroom experiences also came to light. The themes arising from the research are the teachers’ opinions about teaching in FSSs, support from the school and the school management, discipline in the schools, teaching methods, language issues, religion and parent involvement.

**Conclusion:**

It is clear that the specific needs of teachers whose schools were transformed into FSSs will have to be attended to. The teachers are the ones who have to apply inclusive education in their classrooms, and it is essential that teachers’ teaching and personal needs in full-service classrooms are addressed.

**Contribution:**

The article contributes to a better understanding of the teachers’ important role in FSSs and the problems experienced by the teachers. This aligns with the ethos of inclusive education and human rights included in the scope of the journal.

## Introduction

The hail of real democracy in South Africa in 1994 also meant that the equality and the worthiness of each citizen were entrenched in the new constitution. From the side of the government, many attempts were made, and policies were introduced to acknowledge human rights in all spheres of life. This included the terrain of education. Regarding learners and students with disabilities, also referred to as impairments, a new direction that arose internationally was followed and that meant the introduction and acceptance of policies about inclusive education. This direction was new in South Africa and many questions and concerns were raised especially by teachers.

It became clear that although inclusive education was the new way to address the learning and school needs of learners with impairments, many people involved in education and especially teachers had concerns. The continued role of special schools and the introduction of so-called full-service schools (FSSs) were among the many concerns raised.

Teachers are the persons who have to put the policies of the various departments of education into operation. Their acceptance and application of policies are thus pivotal for the new policies to become successful. The Northwest province of South Africa where the research was conducted has four districts and annually an amount of more or less R2 million is awarded to schools to be adapted to become FSSs.

### Full-service schools

Full-service schools were introduced in South Africa to support and enhance inclusive education and the idea was that the FSSs should be in the neighbourhood of learners with barriers to learning and development. According to the philosophy of inclusive education, all learners can learn if the support that they require is provided. Therefore, FSSs should provide support, resources and expertise to any learner who needs it in order to make progress at school. According to the new policy, FSSs would not replace special schools but will provide moderate levels of support (South African Department of Education 2010:21).

It is envisaged that FSSs should be places where diversity and human rights and relations are appreciated and the necessary support systems for all the learners are available (South African Department of Education 2011:4). This could help to minimise the stigma associated with learners with mild physical impairments and with some form of learning problems. Inclusive education was introduced to the education system in South Africa with the implementation of Education White Paper 6, which has since become the official policy. In Education White Paper 6, it is discussed how mainstream schools can be changed to FSSs (South African Department of Education 2005:2–6). In 2012, the Department of Education (DoE) admitted that some of FSSs did not operate as functional FSSs in spite of millions of Rand allocated to FSSs each year (Northwest Department of Education 2012:8).

It is a well-known phenomenon that the people who struggle to implement policies drawn up by the state and its various departments are often not consulted once the policies are implemented.

### Teachers and new education policies

In recent years, many new policies and changes have been introduced in the South African education system. It became clear that, as often happened in the formulation of new education policies, no or little consultation with teachers was done, which is something that occurs in many countries. Gozali et al. (2017) state that although education policies are developed by policymakers and researchers, there is a trend that international organisations such as the United Nations Educational, Scientific and Cultural Organization (UNESCO) and the World Bank could play a role in driving the direction of education:

‘Yet while the importance of mobilizing all stakeholders has been long acknowledged, the voices of researchers, policymakers, and international organizations continue to dominate the scene, while the voices of teachers, the most important school input influencing student outcomes continue to be neglected both in literature and in policy-making.’ (p. 32)

How well these new policies and changes in the education system work have to be researched as this is the only way in which the success of the new policies can be assessed. In the school system, teachers are on the frontline to apply the new policies and changes, but there is a lack of research about teachers’ opinions about the viability of new policies. In this article, the experiences of teachers in FSSs in one province of South Africa will be discussed. It provides insight into the opinions, albeit on a small scale, of a group of teachers teaching in FSSs in a province in South Africa. It is an attempt to let the voice of teachers in FSSs be heard so that the successes and failures of FSSs could be brought to the light and, where necessary, changes in the policies about FSSs could be introduced.

## Research methods and design

In the research discussed in this article, a qualitative approach was used to collect the data because a qualitative approach provides researchers the opportunity to be sensitive to the human world. Researchers’ own humanity helps them to understand and interpret what the participants convey, as well as their circumstances (Babbie & Mouton 2007:50). Qualitative research can elucidate what happens in true-to-life situations such as teachers’ interactions at school (Denzin & Lincoln 2020:3). Leech and Onwuegbuzie (2007:558) also state that qualitative research is very useful to obtain insight into problematic or difficult experiences of people and the significance that individuals attach to the experiences.

### Data collection

In this research, informal observations written down as field notes and focus group interviews were used. Using triangulation, it can be ensured that rich and comprehensive information could be obtained. When triangulation is used, the phenomena in the research could also be understood more fully (Leech & Onwuegbuzie 2007:557). This helped the researchers to not only capture information about the opinions and experiences of the teachers but also information regarding their emotions and feelings.

The participants of this research were 20 teachers from four FSSs in a province in South Africa. Purposive sampling was used as the teachers were selected based on their teaching experience in FSSs and thus the researchers believe that the selected group of teachers could provide a richness of knowledge. This aligns with Sargeant’s (2012:2) opinion that qualitative research helps researchers to purposely select participants who will help them to understand the research topic. Focus group discussions were conducted, and field notes were made during observation, which provided the opportunity to capture the participants’ emotions and body language as well.

Four focus group discussions were conducted, and each focus group consisted of five teachers. There were 16 female and 4 male participants, and they taught Grade R to Grade 7. The discussions took more or less an hour depending on the eagerness of the participants to share their opinions. Each participant was observed, thus 20 classroom visits were completed. The classroom observations were planned for half an hour, but sometimes it was longer. There were many types of impairments in the classrooms such as hard of hearing and physical and visual impairments. Some teachers indicated that there were learning problems arising from social problems and when children were severely neglected at home. There were also learners with dyslexia, dyscalculia and dysgraphia resulting in learning problems. About 10% of the learners suffered from visual, hearing and physical impairments; about 60% of the learners had some form of learning problem caused by dyslexia, dyscalculia or dysgraphia. In the opinion of the teachers, there was quite a big group of learners who had learning problems caused by child neglect and severe poverty.

It was estimated after the classroom observations that about 30% of the learners were well-supported and that they were responsive. The rest of the learners were quite disruptive, and it seemed as if they did not know or understand what to do.

The provincial Department of Education, the principals of the four schools and the teacher participants provided written permission to conduct the research. The authors kept the data and they were the only persons who had access to it. The institution to which the authors are attached provided an ethical clearance certificate.

The conceptual framework underpinning this research comes from the interpretive practice, which allows researchers to understand social phenomena in the milieu in which they are constructed and repeated through activities (Akerlind 2005:323). Interpretivism departs from the view that insight into reality is obtained through social constructions, which include language, opinions, tools and documents (Giorgi & Giorgi 2003:71). This was used to help the researchers to understand the participants’ unique acceptance, teaching skills, training and organisational issues regarding FSSs and inclusive education. Interpretivism also guided the researchers to see the teachers’ unique situations and emotions from the perspective of the teachers as the human actors in the classroom ‘drama’ (Chowdbury 2014:433).

### Data analysis

A narrative analysis was followed during which the participants’ opinions and experiences were transcribed. After this, data were arranged into core categories. Themes arising from the field notes taken during classroom observation were also identified. This provided the opportunity to capture especially some participants’ feelings and emotions. All the data were then organised into themes and in some instances into subthemes. The researchers used triangulation to include data from more than one data collection method.

### Ethical considerations

Ethical clearance to conduct this study was obtained from the University of South Africa College of Education Research Ethics Committee (No. 2014/11/17/06926916/33/MC).

## Results

The main purpose of the research was to investigate the attitudes and opinions of teachers in FSSs about the success and the challenges in the application of policies in these schools. The data presented nine main themes, and under one of the themes, support from the school and the school management, three subthemes emerged.

The participants, whose direct quotations are used in this article, formed part of the research for a doctoral study (Mobara 2018). All of them were professionally trained teachers. Most of the participants had more than 10 years of teaching experience. There was one teacher with 33 years of teaching experience, one with 35 years and one with 40 years.

### Theme 1: Teachers’ negative attitudes to full-service schools

It should be kept in mind that most of teachers at FSSs did not choose to teach at FSSs. Many of them were already employed at schools when these schools were changed into FSSs. These teachers and their principals simply had to accept the change based on new policies from the then Department of Education and had to welcome learners with minor barriers to learning and development in their schools.

During the interviews, it became clear that there was a group of teachers who had accepted the fact that they had to deal with inclusive education in their schools and they supported the learners with barriers to learning in their classrooms. There were, however, some teachers who were participants in this research had their reservations about the implementation of Education White Paper 6 and about the introduction of FSSs. Most of the reservations relate to the school and the classroom situation, but there were also concerns about the involvement of parents.

The negative attitude to inclusivity is apparent in the following statement of one of the participants: ‘I am not comfortable with inclusive education. No, it is just extra work’ (P2, female, teacher).

A group of the participating teachers were indifferent to the problems of learners with barriers to the language of teaching and learning. One of the participants simply rolled his eyes and said that it was another story.

When the issue of learners with reading problems was raised during the group discussions, one participant shouted: ‘These learners drive me crazy. I chase them out of the class if they cannot read’ (P8, female, teacher).

It seemed as if some of the participants could not control their emotions as one of them said about a boy who could not read: ‘He either sits there, as I don’t want to see him, or he goes home. I could not be bothered. As long as he is out of my sight’ (P11, male, teacher).

### Theme 2: Acceptance of the ethos of inclusive education

Not all the points of view at the four selected schools were negative and the one researcher wrote in her field notes gathered during observation that there were many positive issues with regard to inclusive education at the four schools. Three of the 20 teachers were of the opinion that a FSS was a good idea. They felt that a FSS allowed learners to stay in their communities and the learners did not have to go to a hostel. Four teachers in different schools commented on how learners with special needs in the FSSs had changed the mindset of some parents and community members. During group discussions, a few teachers pointed out that some of their community members showed more empathy towards learners with special needs.

During observation there were specific incidents when learners with special needs were supported by the teachers. A learner who was hard of hearing was helped by a teacher to replace the battery of his hearing aid. The teacher of a learner who was struggling with reading went back to using a board game to emphasise single phonics. Another teacher used concrete objects so that a learner with dyscalculia could grasp number concepts. There were two teachers who provided two of the learners in their classes with additional time to complete their sentences. A teacher read the questions that had to be completed to a learner who could not read.

There were also a few incidents where the authors noticed that learners accepted and supported their classmates with special needs. During observation, for instance, one learner got up from his seat and went to another learner to assist him by turning his book so that he could write on the right side. Outside the classrooms it was observed that there was another learner who was assisting a classmate with his wheelchair.

### Theme 3: The influence of class size

The recommended learner-to-teacher ratio in schools in South Africa is 30:1. According to the School Realities Publication, EMIS, in 2022, the learner-to-teacher ratio on national level in South African schools where teachers are paid by the state was, however, 34:1 (Careersportal 2022). As far back as 2012 research in the United Kingdom showed that smaller class size had a positive effect on school success. In smaller classes, teachers had the opportunity to focus on individual learners (Class size and education in England evidence report 2012:2).

In some of the classes that were observed in the research under discussion, it was evident that there were too many learners in some of the classes and this made it impossible for the class teachers to attend to the specific and special needs of learners with barriers to learning and development. In one Grade 1 class, there were, for instance, 50 learners; in a Grade 2 class there were 45 learners and in a Grade 3 class there were 52 learners. In the opinion of the researchers, this was detrimental to the enhancement of inclusive education in some classes.

### Theme 4: Support from the school and the school management to enhance the ethos of full-service schools

Certain issues arose that were related to support from the school and the school management. These issues will be discussed under the following headings: the principal, the school management team (SMT) and the school-based support team (SBST).

#### The principal

All staff members should have the opportunity to attend further training and so develop themselves professionally. It is in the first place the duty of principals to see to it that all staff members get a chance to go for training because ‘[e]ffective principals also encourage continual professional learning’ (The school principal as leader: guiding schools to better teaching and learning 2013:11). Principals should also keep record and track of the teachers’ needs for professional development (The school principal as leader: guiding schools to better teaching and learning 2013:15).

This caused the teachers to become stressed. A participant said that the principal and the SMT did not assist the teachers at all. One participant said: ‘Our principal is not interested in anything regarding inclusive education. He said that I should go and teach my learners in class and stop troubling him with inclusive nonsense’ (P19, female, teacher).

In the field notes it was written that the body language and gestures of the participant reflected her helplessness and that she could see that the teacher participant was becoming demotivated. During the focus groups, the emotional stress of the teacher participants also became clear and one participant made the following remark about her principal: ‘Our principal is not interested in anything regarding inclusive education. He just says do what you have to do’ (P15, female, teacher).

From a research project conducted in Gauteng, one of the provinces of South Africa, it becomes clear that principals are also in need of inclusive education training. One of the participating principals in this project said, for instance:

‘As principals we need training, we really need training on this really because we talk inclusion, inclusion means different things, different problems that needs to be addressed. How as a principal am I going to know how the teachers must support the child in class?’ (Mahlo 2011:162)

An effective principal is the ‘broker’ of conditions at his or her school. Good principals are persons who can set an example showing that with a positive attitude things can be done in a school and thus in the case of full-service can lead to show the way how inclusive education can work. By creating a positive culture at school, children should be able to learn (Leadership matters 2013).

#### The school management team

At three of the four schools, the participants indicated that they were not helped by the members of the SMT to apply inclusive education and to support all learners in their classes.

The curriculum that has to be completed was a serious issue for some participants. They did not know whether they should concentrate more on supporting learners with barriers to learning, which is time-consuming, or whether they should try to finish the curriculum, which could mean that some of the learners with barriers to learning would be left behind. A participant said: ‘We have to finish the curriculum and complete the number of activities prescribed by DoE’ (P9, female, teacher).

The participants explained that they did not get any assistance from members of the SMTs and that they had to handle the big task of teaching learners with barriers alone. One participant cried out: ‘Nobody listens to us. We are voiceless. We have problems in our classes. We are struggling with learners who need additional support. We can’t help them’ (P18, female, teacher). The field notes captured the anxiety of this participant, which was very obvious.

Teachers at FSSs should receive training to apply an inclusive education strategy called Screening, Identification, Assessment and Support (SIAS) (South African Department of Education 2008:1–2). The training could be helpful to apply inclusive education strategies in their classrooms, but at the schools that formed part of the research, it did not happen. At one school a participant remarked: ‘We don’t know what SIAS is. How can we use it, we were not trained on it?’ (P16, female, teacher). At another school a participant opined about the training in SIAS: ‘SIAS – No it was not training, it was only orientation, and it is too difficult to complete. There is too much paperwork’ (P11, female, teacher).

At one school a participant took the issue further when she said that when colleagues from her school went for in-service training, no feedback about the training was provided to the rest of the staff. She wondered if the colleagues who went for training saw it merely as a trip and not as a chance to develop professionally.

When one considers the serious issues mentioned by the participants relating to their principals and the members of the SMTs, it becomes evident that the practical implementation of the opening of FSSs in South Africa as set out in White Paper 6 should ring alarm bells in the various departments of education.

#### School-based support teams

In some of the schools in this research, there were SBSTs, but the participants complained that they were not functional. In some schools there were even files that were neatly covered, but it was all window-dressing. A participant reiterated the following: ‘All the files are smartly covered, but not any of the learners I referred to the SBST are ever helped’ (P8, female, teacher).

No learners or teachers were supported by the SBSTs. A group of the participants said that the members of the SBSTs seem to lack the necessary knowledge and experience as they relied on individual teachers’ expertise and experience at their schools to provide them with assistance to help learners with barriers to learning. One participant frankly said: ‘We have a SBST at our school, but they can’t help our learners’ (P20, female, teacher). According to the participants, there were also cases where learners with barriers to learning were simply ignored by the SBSTs.

In research about FSSs in the Gauteng province of South Africa, one of the participants stressed the fact that if a SBST is functional at a school, inclusion will work. In the responses of some participants, it became evident that there is a strong belief that if the SBSTs are functional at schools, then inclusion will work. One respondent said: ‘If [*a*] SBST is functional then I[*nclusive*] E[*ducation*] will work, because the SBST it receives, or it gets all the conditions that needs to be addressed’ (Mahlo 2011:163). This was confirmed by another respondent: ‘The SBST if functional, that is a step towards the implementation of I[*nclusive*] E[*ducation*] you can hear from what I have already said’ (Mahlo 2011:163).

As far back as 1994, Bronfenbrenner and Ceci (1994:572) said that people act according to their experience. In the research under discussion, it became clear that teaching at a FSS was an experience that some of the participants did not enjoy. These participants became demotivated because they did not get the support that they needed from the SMTs at their schools. Some of them felt that they want to stop teaching or wanted to go on long sick leave. One of the participants said the following about her health: ‘These children affect my health, my blood pressure’ (P14, female, teacher).

It is clear that a policy that changes ordinary mainstream schools into FSSs, thus placing learners with different abilities in the same classrooms, does not mean that inclusion is reached. It is necessary to change the whole school, the teachers and also the school community so that learners with different learning needs could be accommodated (Mariga, McConkey & Myezwa 2014:20).

### Theme 5: Discipline in the schools

In the South African Constitution (1996), there are various rights that protect learners from corporal punishment. Section 10 acknowledges, for instance, the right of all citizens to have their dignity protected. In Section 12(1), the right to freedom and security is protected.

It includes rights such as to be free from all forms of violence – not to be tortured or to be treated or punished in a cruel, degrading, or inhuman manner. In Section 28(1), specific rights of a child are included. Every child has the right to be protected against maltreatment, neglect, abuse or degradation.

All forms of corporal punishment of children in South Africa was effectively banned on 18 September 2018 by the Constitutional Court of South Africa. In the judgement, the research that was performed was highlighted. That research showed that all corporal punishment has the potential to be damaging and could be part of a wider circle of violence. The research also showed that there are existing alternative nonviolent methods that could be used to raise a child to become a responsible member of society and that it was in the best interest of a child to stop defending moderate chastisement (South Africa prohibits all corporal punishment of children 2019).

Some of the teachers who participated in the research complained about certain learners who disrupted their classes and the teaching that should take place. This is understandable because learners with learning problems caused by, for example, attention deficits are also admitted to FSSs and often these learners are disruptive in class. Some of the participants explained that they were at a loss about what to do with undisciplined learners and one said:

‘We as teachers feel threatened at times by the learners. They misbehave and no one knows what to do with them. We just leave them alone. They can do their work or leave it. We don’t assist them as their parents don’t want to help us discipline their children.’ (P15, female, teacher)

It was clear that some participants did not know how to handle such disruptive behaviour as the only form of punishment that they knew was corporal punishment. The participants complained that although they were aware of the new policies about punishment and discipline, there were no practical guidelines on how to deal with discipline issues in their classrooms.

### Theme 6: Teaching methods

During the visits to the FSSs, it became clear that the members of staff were grateful for the additional resources that the schools received, which included ramps and assistance devices.

Quite often the additional resources were not used at all because the existing professional development programme does not make provision to really support teachers and other members of staff to implement inclusion in its true sense. According to some of the teachers at the FSSs’ in a 2012 document of the Northwest Department of Education it was admitted that the teachers at FSSs had problems to adjust their teaching. In this document it is stated:

‘[*I*]t is the lack of reasonable adjustments to their teaching methods, the use of extra equipment and teaching aids and the improvement of learner performance that they experience as a problem.’ (Northwest Department of Education 2012:4–5)

Regarding teaching methods, Voltz, Brazil and Ford (2001:28) opine that a single method is never suitable when there are learners with diverse abilities and learning needs in one classroom. Teachers should use a variety of methods and teaching materials to cater for the diversity in a classroom. In his research at an FSS, Ntsoaki (2017:42) affirms this view reporting that a participant said that a teacher had to try quite a number of teaching methods when there are learners with diverse abilities and learning needs in his or her class. A teacher in a FSS cannot use one teaching method only.

### Theme 7: Language issues

Regarding language teaching, Wildsmith-Cromarty and Balfour (2019:296) say that often learner success depends on the ability of teachers to develop learners’ languages for literacy and for language of learning and teaching (LoLT) purposes. The authors opine that research has shown that if literacy is developed in young learners’ home languages, it will have a positive effect on the literacy development in the LoLT, should it differ from the home languages.

Owen-Smith (2010:31) goes a step further when she states that learners are disadvantaged if they cannot use the language that they are most familiar with, which is usually the home language, at school. It is unsure if these learners will be able to perform to the best of their ability at school. This could weaken the learners’ self-confidence and may result in underachievement. By law no learner in South Africa may be refused to be admitted to a public school because of unfair discrimination or unequal treatment based on race, language or religion (South African Department of Education 2006:5).

In his research at an FSS, Ntsoaki (2017:44) reports that the LoLT of some learners was a problem. The LoLT of the school was English, but learners who did not speak English were admitted. This meant that quite often and when necessary, the teachers had to code switch. One participant said that at the school ‘language is a serious barrier’ (Ntsoaki 2017:44).

The teachers who were the participants in this research had learners who did not understand or speak the LoLT of the school. In spite of this, the participants were expected to teach these learners and to complete the curriculum. For some of the participants this was a nightmare.

One participant who was using Setswana (one of the official languages of South Africa) said regarding a learner who did not speak Setswana about the management of the school and the curriculum specialist: ‘They will not ask or understand that the learner does not understand Setswana. I will be in *big big* [*italised for emphasis*] trouble’ (P2, female, teacher).

### Theme 8: Attitude to different religions

During the research an issue concerning learners’ religious practices arose. It is a general practice in South African schools that children with a Muslim background usually attend schools where Muslim is the dominant religion. Attending mosque early on a Friday afternoon is thus not a problem. In the FSSs included in the research, there were a few Muslim learners and they had to leave early on a Friday. This meant that these learners missed some classes every Friday afternoon and the teachers had to make provision for the learners to catch up with the class work done while they were absent. This gave rise to another need of learners that the participants had to attend to.

Some of the participants were frank when providing their opinions about learners who came from denominations other than their own. One participant said:

‘Don’t know why a person must struggle with these different types of learners! Now I must struggle with this child having to carry their religious beliefs. Do I look like a pastor?’ (P3, female, teacher).

In the field notes the agitation of another participant about the issue of religion was noticed because she asserted during the group interview:

‘Why does this boy need to attend mosque on a Friday? He is leaving school every Friday. It becomes my problem as he’s always behind with his work. I have to assist him to catch up with his work. I refuse; I do not want to do it.’ (P3, female, teacher)

The two participants were rather upset and troubled when one considers their answers. It was evident that they were not prepared during in-service training or by the management of the schools about cultural changes that might occur with the introduction of inclusive education and FSSs.

It was also clear that some of the participants were prejudiced. Prejudice should not be allowed in schools under the auspices of the government let alone FSSs. Regarding this, Hodgson (2017) states:

‘Although the state should promote all religions, it is required to do so without any favour for or prejudice against any particular religions or beliefs. It is particularly important for minority religions and cultures …’ (p. 188)

It is also not in line with what is expected of teachers according to the South African Council for Educators (SACE) because teachers have to ‘acknowledge the uniqueness, individuality and specific needs of each learner, guiding and encouraging each to realise his or her potentialities’ (SACE 2008:12–13). The remarks of the participants were, however, also a reflection of their frustration as it was evident that they were not sufficiently prepared to attend to the diverse needs of the learners in their classrooms.

### Theme 9: Parent involvement

Research elsewhere and in South Africa often reveals how important parental involvement is for young learners’ success at school and for their well-being. Involvement of parents enhances learners’ self-esteem, it provides the opportunity for parents to get to know the teachers, which could be useful to facilitate their children’s progress, and it also helps to develop a positive attitude to school (Yu & Shandu 2017:160). Ntsoaki (2017) reports that in his interviews with teachers at an FSS, the participants informed him that the parents were not supportive and involved in their children’s teaching and learning at the school:

‘We are sitting on a serious problem here as many parents do not give their children support at home. When we give learners homework, most of them come back to school without doing it.’ (Ntsoaki, 2017:42)

It seems as if a lack of parental involvement is a recurring theme in research about schools and also FSSs in South Africa and it also surfaced in the research discussed in this article. When parents are requested to come to school, the participants complained that they did not come. One participant explained:

‘When we have learners with behaviour problems, and write letters, we call their parents by telephone, and we send messages. We need to see them. They don’t respond, they are nowhere to be found, there is no response from parents.’ (P7, female, teacher)

This was affirmed by another participant:

‘Parents are not interested in the code of conduct of the school. They expect the school to take full responsibility of their child, and they as parents don’t even want to help with the discipline.’ (P13, female, teacher)

There were also complaints about parents who become aggressive as one participant explained:

‘I was slapped in my face by a mother. I sent the learner home to go and call her mother as she said she was at home. I was teaching reading on the mat with a group of learners, when the classroom door opened and next thing [*participant sobbed again*], I felt pain on my face. Now I just leave learners if their parents don’t care, why must I worry?’

In the FSSs that formed part of this study, many parents also did not respond when the teachers requested them to give their consent that the school could apply for additional support for learners with behavioural problems or other barriers to learning. The parents simply did not come to the schools. This also meant that the teachers could not get the parents’ consent to apply for additional support for their children.

## Discussion

When one considers the feedback from the participants in the research, it is evident that they experienced many problems teaching at FSS. The difficulties that the participants mentioned during the research can be considered on two levels of human functioning: the knowledge or cognitive level and the emotional level.

On the cognitive level, we as the researchers realised that most of the participants lacked knowledge on how to adjust their teaching methods according to the learning needs of their learners and they also did not know how to use the extra equipment and aids provided to the FSSs by the provincial Department of Education. Some participants mentioned that although they know the SIAS document, they were not trained on how to use and apply it in their schools. There was a complaint that only some staff members went for in-service training but no feedback about the training was given to the other staff members afterwards. We opine that the use and classroom application of the SIAS document could help teachers and school management to apply the ethos of inclusive education in FSSs. This could only be realised if all staff members in the FSSs receive in-service training and specific training to apply the SIAS document in the classroom. The opportunity to teach all learners according to their specific learning needs was thus not realised in these FSSs. It was evident to us that the participants knew the new law about corporal punishment in schools but nobody helped them with strategies to maintain discipline.

Some of the participants realised that parental involvement is an important contributing factor for children’s success at school. They did, however, not know how to get the parents involved in the school and in the schoolwork of their children and they were not supported by the SMT to get parents involved. The issue that principals should receive training regarding inclusive education was also evident. A group of the participating teachers mentioned that the members of their SBSTs lacked the necessary knowledge to help them with teaching and disciplinary issues in their classrooms. Some participants opined that they did not know whether they should try to teach the full curriculum to the learners in their classes or if they should ‘waste’ time trying to support all the learners according to their learning needs. It seems as if class size in FSSs should be an issue that needs the urgent attention of the National Department of Education.

On the emotional level, rather disturbing issues arose during the research process. This is when, especially in the field notes, the unspoken voice of the participants was captured. Interpretivism helped the researchers to better understand the teachers as the human actors in the classrooms of FSSs. We opine that a lack of support from the SMT was the predominant cause that some participants became demotivated. Emotional stress was evident among a group of participants, and this was what we attributed it especially to the attitude of the principal and SMT. We observed that some participants appeared to be anxious because they felt that nobody was listening to them and that they were actually voiceless. Agitation and frustration were also deduced from some of the responses. The researchers interpreted the comments about learners who have to attend mosque on a Friday and thus be taught additionally as prejudice, which is something that has to be avoided in any education system. We are of the opinion that the emotional issues that became evident during the research process could have a negative impact on the health and well-being of the participants who teach in FSSs.

Any change in the education system including inclusive education should not merely be a wonderful theoretical plan; it should also address the practical application and implications of such change. First and foremost, it is the duty of the principal and the SMT in FSSs to make sure that teachers are prepared for inclusive education in their classrooms. Teachers should be trained to apply different methods if the needs of their learners necessitate it. They will have to be well informed about changes including cultural changes in their classrooms and in their teaching.

Teachers who have to carry the responsibility to practically apply the spirit of inclusive education in the schools and in their classrooms should be consulted when the policies are planned, and they should also be constantly supported to apply the policies. The well-being and the health of teachers in FSSs who have to carry the responsibility to have learners with identified barriers to learning in their classrooms should be attended to. It is rather sad that teachers at FSSs should have such negative views about teaching learners with barriers to learning and that they should have reservations about the viability of applying the ethos of inclusive education in their classrooms.

A group of the FSS teachers who were the participants in this research voiced their predominantly negative opinions about teaching in FSSs. This could be reason for their reservations about the success of putting Education White Paper 6 into practice. Although the research was conducted in one province, it is apparent that some of the participants view teaching at an FSS as a nightmare because they did not receive the necessary training and support to teach learners with diverse learning needs. It is evident that the teachers who have to put the changes into practice in FSS classrooms should be considered and therefore the ‘voice’ of teachers should play a vital role in any education system and especially in inclusive education.

It was encouraging to hear a positive ‘voice’ about inclusive education expressed by a few of teacher participants. This could be used to grow a positive attitude towards inclusive education among the parents and community members. Learners who help their classmates could be acknowledged in some or other way.

## Conclusion

We conclude that the specific needs of teachers whose schools were transformed into FSSs are the result of challenges in the education system. The teaching needs of teachers in FSSs and their emotional well-being will have to be addressed. However, teachers in these schools should also remember to adhere to the requirements of human rights in all situations and do so consistently. The positive attitude about inclusive education expressed by some teachers could be channelled to change their colleagues’ attitude regarding learners with special needs. In the end, the teachers are the ones who have to apply inclusive education in their classrooms and therefore it is crucial that their ‘voice’ should be heard so that FSSs in South Africa can be successful.
